# Transient Diabetes Insipidus After Vasopressin Discontinuation

**DOI:** 10.7759/cureus.61253

**Published:** 2024-05-28

**Authors:** Audrey L Chai, Rastko Rakočević, Firas M El-Baba, Keith Killu

**Affiliations:** 1 Department of Medicine, University of Southern California, Los Angeles, USA; 2 Division of Pulmonary, Critical Care and Sleep Medicine, Department of Medicine, University of Southern California, Los Angeles, USA

**Keywords:** vasopressor, hypernatremia, polyuria, vasopressin, shock, intensive care, diabetes insipidus

## Abstract

Vasopressin infusion is commonly used in intensive care settings during states of advanced vasodilatory shock for its vasoconstrictive properties. Vasopressin also acts on renal tubular cell receptors in the collecting ducts of kidneys to allow for water reabsorption. The sudden discontinuation of vasopressin infusion can lead to the development of transient diabetes insipidus (DI) with classic findings of polyuria, dilute urine, and hypernatremia. We report the case of a 59-year-old male who underwent an emergent bedside cricothyrotomy procedure secondary to papillary carcinoma of the thyroid and subsequently developed septic shock requiring initiation of vasopressin infusion for hemodynamic support. He remained on vasopressin for five days before the infusion was discontinued after clinical improvement. Within 12 hours of vasopressin discontinuation, the patient developed polyuria (> 3 L/day urine output) with volumes as high as 1 L per hour. His serum sodium levels increased more than 10 mmol/L from 137 to 149 mmol/L. This case is unique from prior reports, as our patient was without any neurological or neurosurgical comorbidities that would predispose him to an organic central cause of DI. Furthermore, the patient’s large-volume diuresis and serum abnormalities spontaneously self-improved within 24 hours without significant medical intervention. In conclusion, this case adds to a growing number of reports of transient DI following vasopressin withdrawal, demonstrating the need to formally recognize this occurrence as a potential consequence of vasopressin use in intensive care settings.

## Introduction

Vasopressin is an endogenous hormone released from the posterior pituitary gland that acts on multiple receptors in the body to regulate crucial physiologic processes, such as osmoregulation and vascular smooth muscle contraction [1]. During shock states, there is an initial rapid release of vasopressin stores from the posterior pituitary gland. The subsequent rise in vasopressin levels leads to a powerful vasoconstrictive effect [2]. As the shock state continues to evolve, vasopressin stores are depleted, resulting in a relative deficiency. The therapeutic infusion of exogenous vasopressin is commonly used in the treatment of vasodilatory shock [3]. The hypotension of septic shock in particular is thought to be due to a deficiency in plasma vasopressin levels, leading to the established role of vasopressin in the treatment of vasodilatory septic shock [4,5]. Compared to those with other shock states, patients with septic shock have also been shown to have relatively lower serum vasopressin levels [6,7].

Diabetes insipidus (DI) is a syndrome characterized by an impaired ability to reabsorb water, resulting in the excretion of large volumes of hypotonic urine, hypernatremia, and volume depletion [8]. DI has classically been further characterized into subgroups, including central DI from deficient secretion of vasopressin, nephrogenic DI due to loss of sensitivity of the kidney to antidiuretic hormone, and gestational DI due to production of vasopressinase during pregnancy [8,9]. Recently, there have been case reports of transient DI (tDI) following the withdrawal of vasopressin infusion for the treatment of shock [10]. Initial case reports included patients with acute neurological or neurosurgical disorders, such as ventriculoperitoneal shunt, traumatic brain injury, or subarachnoid hemorrhage. tDI is more common in these patients due to direct injury to the hypothalamus, pituitary gland, or blood supply [11,12]. To date, there have been few case reports describing this phenomenon in patients without any underlying neurological pathologies [10,13]. Here, we present the case of tDI in a patient following the discontinuation of vasopressin for treatment of septic shock after an emergent surgical airway procedure.

## Case presentation

A 59-year-old male, with a past medical history of previously treated papillary thyroid cancer, was admitted to the hospital step-down unit for surgical evaluation and management by otorhinolaryngology for a large neck mass with tracheal involvement concerning recurrent cancer. Soon after admission on hospital day (HD) one, the patient’s breathing became progressively more dyspneic with stridor, and he experienced concurrent oxygen desaturations requiring increasing levels of oxygen support from the nasal cannula to a non-rebreather mask. He was transferred to the intensive care unit (ICU) and ultimately required an emergent bedside cricothyrotomy procedure for airway securement given extensive tumor involvement of the upper airway, limiting orotracheal intubation. Throughout this period, there was no significant hypoxemic event to cause brain injury. During and following resuscitation, the patient became hypotensive, requiring the initiation of norepinephrine and vasopressin infusions for hemodynamic support.

The patient’s shock was thought to be secondary to hypovolemia due to fluid and blood loss, as well as vasodilation from the inflammatory response after extensive tissue manipulation during the emergent bedside procedure. Possible aspiration and pneumonia could have also caused sepsis and shock. The patient was empirically started on broad-spectrum antibiotics (piperacillin/tazobactam and vancomycin). He was appropriately fluid resuscitated but remained in a shock state. Other medications the patient received at this time included high-dose steroids (intravenous dexamethasone, newly started after admission), levothyroxine, lactulose, senna, prophylactic subcutaneous heparin, and prophylactic intravenous pantoprazole. The patient was maintained in a sedated and paralyzed state with an endotracheal tube secured in the surgical airway before he was taken to surgery on HD four for a definitive tracheostomy. Post-operatively following surgical tracheostomy, he remained on norepinephrine and vasopressin infusions. Vasopressin was being infused at a rate of 0.04 units/minute until HD five when his blood pressure improved, and the vasopressin was discontinued.

**Figure 1:**
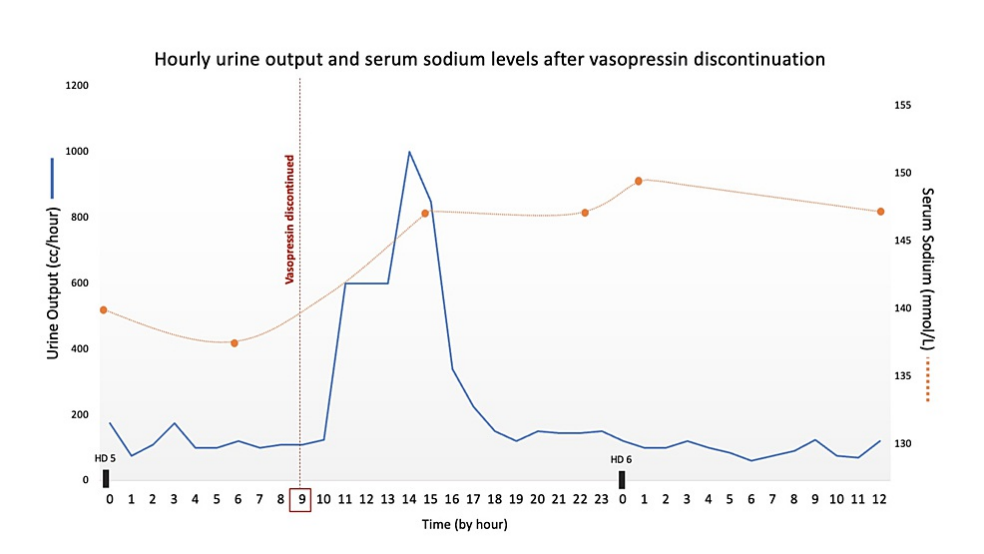
Hourly breakdown of the patient’s urine output and serum sodium levels on hospital days 5-6 before and after vasopressin infusion discontinuation at 09:00 on hospital day 5.

Over the 12 hours following vasopressin discontinuation, the patient’s urine output was noted to significantly increase from an average rate of 50-200 cc/hour to 350-1,000 cc/hour (Figure 1). In total, the patient’s urine output was 4.9 L (average output of 408 cc/hour) over the 12-hour period following vasopressin discontinuation on HD five. Additionally, chemistry results showed a swift rise in serum sodium from 137 to 149 mmol/L (Figure 1). There was no significant rise in his serum creatinine level to indicate any evidence of renal dysfunction. The patient had not been given any diuretics in the preceding 72 hours, his point of care glucose levels indicated euglycemia, and thyroid hormone studies were within normal range. Urine studies were obtained, revealing a low urine osmolality of 172 mOsm/kg and urine sodium of 23 mmol/L. Serum osmolality was elevated at 307 mOsm/kg (normal range: 275-295 mOsm/kg).

Enteral-free water (200 cc tap water every four hours) was started for the hypernatremia, and other electrolytes were replenished to goal levels in the setting of the patient’s increased self-diuresis. Within 24 hours, the patient’s urine output spontaneously returned to normal rates, and serum sodium levels began downtrending without other interventions or the need for additional medication administration. Vasopressin never needed to be restarted, and the patient’s sodium and urine output levels remained normal. The patient displayed no abnormal neurological signs throughout this period and awakened appropriately after sedation was weaned. The patient’s clinical status ultimately improved, and he was transferred out of the ICU and eventually discharged home.

## Discussion

Following the withdrawal of vasopressin infusion, this patient experienced significant hypotonic polyuria with an associated increase in serum sodium levels consistent with DI. Though this rare phenomenon has been described before, this report is unique from others as our patient did not have any neurological abnormalities, and he experienced rapid self-resolution of polyuria before any intervention such as desmopressin was given.

DI is a common occurrence in ICU patients in the settings of traumatic brain injuries, pituitary surgeries, brain death, and other insidious cerebral diseases that lead to loss of antidiuretic hormone (ADH) secretion [9]. The earliest case reports of tDI following vasopressin withdrawal included patients from neurocritical care units with underlying neurological pathology and recent neurosurgical procedures. The first reported case was of a patient who had a history of viral encephalitis and hydrocephalus that required ventriculoperitoneal shunt placement. He re-presented to the hospital for fever, hypotension, and respiratory failure due to pneumonia requiring ICU admission for presumed septic shock and vasopressin infusion. Discontinuation of the vasopressin infusion was followed by significant diuresis (8.4 L) and rapid-onset hypernatremia (serum sodium concentration increased from 132 to 157 mEq/L over eight hours). The diuresis could only be explained by inappropriate water diuresis, and when vasopressin infusion was restarted, the diuresis ceased [11]. The first case series of tDI after vasopressin discontinuation included six neurosurgical patients who had varying primary brain pathologies including brain tumors, cerebral vascular abnormalities, and cerebral hemorrhage events. A review of the patient’s records and laboratory values in this series showed recurrent elevated serum sodium and urine output and decreased urine specific gravity after discontinuation of vasopressin. Interestingly, the resumption of vasopressin or administration of desmopressin led to resolution of tDI [12]. These cases demonstrated the classic findings of hypernatremia, polyuria, and resolution of tDi after desmopressin infusion.

Outside of the classic neuro-ICU patient population, there was one recent report of this tDI phenomenon occurring in a medical ICU patient: a 52-year-old man with COVID-19-related acute respiratory distress syndrome required initiation of veno-venous extracorporeal membrane oxygenation and infusion of vasopressors, including vasopressin, for treatment of shock. The continuous use of vasopressin over the course of five days in this patient was thought to have inadvertently shut off the antidiuretic hormone production in the hypothalamus and thus disrupted the hypothalamic-pituitary axis and transport of ADH to the kidneys [13].

Other potential etiologies for our patient’s sudden increase in urine output and hypernatremia were considered: He had no intra-cranial pathology seen on neurological imaging. He had no other significant medical comorbidities. He did not experience any urinary obstruction, and his renal function tests (serum creatinine and blood urea nitrogen levels) were normal. He had no history of diabetes, and his glucose levels were at goal. He was not receiving any other medications or intravenous fluids that would have led to polyuria. His thyroid function labs were within normal limits. Thus, it is thought that the sudden withdrawal of vasopressin led to the loss of antidiuretic effect and the formation of large-volume dilute urine, representing a transient form of DI. Potential physiological explanations for this development include downregulation of the vasopressin V2 (V2R) receptors on the renal tubular cells of the collecting ducts and negative feedback on the posterior pituitary gland [14].

## Conclusions

The first reports of tDI following vasopressin cessation described the event as a rare phenomenon; however, this current case presentation adds to a small but growing body of literature demonstrating that this occurrence may be more common than previously thought. There is now mounting evidence to suggest that temporary DI and the associated polyuria and electrolyte disturbances could be consequences of vasopressin withdrawal. Providers should take these potential complications into consideration and have a low threshold for monitoring patients’ urine output and serum and urine laboratory upon discontinuation of vasopressin infusion. Further studies are required to determine risk factors for developing tDI, identify vulnerable patient populations, and detail the pathophysiology behind this phenomenon.
